# Ophthalmoplegia starting with a headache circumscribed in a line-shaped area: a subtype of ophthalmoplegic migraine?

**DOI:** 10.1186/1129-2377-15-19

**Published:** 2014-04-16

**Authors:** Yu Wang, Xian-Hong Wang, Miao-Miao Tian, Cheng-Juan Xie, Ying Liu, Qing-Qing Pan, Ya-Nan Lu

**Affiliations:** 1 Department of Neurology, Epilepsy and Headache group, the First Hospital of Anhui Medical University, Jixi Road 218, Hefei 230022, China

**Keywords:** Recurrent painful ophthalmoplegic neuropathy, Ophthalmoplegic migraine, Migraine, Epicrania fugax, Neuralgia

## Abstract

Recurrent painful ophthalmoplegic neuropathy (RPON), formerly named ophthalmoplegic migraine (OM), is a rare condition characterized by the association of unilateral headaches and the ipsilateral oculomotor nerve palsy. The third cranial nerve is most commonly involved in the recurrent attacks. But it is still debated whether a migraine or an oculomotor neuropathy may be the primary cause of this disorder. Here, we report an elder patient who had a recurrent ophthalmoplegia starting with an unilateral headache circumscribed in an area shaped in a line linking the posterior-parietal region and the ipsilateral eye. And the headache had couple of features similar to that of migraine, such as past history of recurrent migraine attacks, accompaniments of nausea, vomiting, and phonophobia, response to flunarizine and sodium valproate. We may herein report a subtype of OM but not a RPON. This case report indicates that OM may exist as an entity and some OM may be wrongly grouped under the category of RPON in the current international headache classification.

## Background

Recurrent painful ophthalmoplegic neuropathy (RPON), formerly named ophthalmoplegic migraine (OM), is a rare disorder characterized by recurrent headaches and associated palsy of the third, fourth or sixth cranial nerves, with the third cranial nerve most commonly affected. In the first edition of the International Classification of Headache Disorders (ICHD-I) OM had been classified as a variant of migraine [1], in the second edition (ICHD-II), OM had been reclassified under the category of cranial neuralgias [2], while in the third edition (ICHD-3), the term of OM was rejected and has been modified to be RPON [3], basing on the facts that the headache can develop up to 14 days prior to oculomotor paresis, gadolinium enhancement or nerve thickening can be demonstrated using magnetic resonance imaging (MRI), and treatment with corticosteroids is beneficial in some patients [3]. However, the etiopathology of this disorder is still under debate. Some authors propose that migraine is the underlying cause of ophthalmoplegia in this disorder, basing on the analysis of couple of cases [4-6]. Here, we report a patient with recurrent headaches of which the pain was circumscribed in a line-shaped area and had couple of features similar to that of migraine, followed by paresis of the ipsilateral third cranial nerve. Thus, we may herein report a subtype of OM but not a RPON.

## Case presentation

A 63-year-old man came to our department because of recurring headache attacks, sometimes associated with ptosis and diplopia for 2 years. A typical headache attack would start with a mild pain restricted in the right supraorbital margin point and almost immediately after the onset of this pain the patient would suffer a distinctive head pain in the ipsilateral side. The head pain gradually reached its peak in minutes and was circumscribed within a line-shaped area of 5 mm to 10 mm in width. This line-shaped pain area linked the right supraorbital margin point with the ipsilateral posterior paretial region and was parallel to the sagittal midline of the head. The pain was described as moderate to severe distending, pressure-like and sometimes pulsating sensation with no radiation or moving, and the patient denied this pain epicranial but complain it intracranial. The headache was usually associated with nausea and phonophobia, often precipitated by physical activity and would last for 2 to 3 days and remitted spontaneously. In recent two years, the patient has had more line-shaped headaches without ophthalmoplegia, occurring approximately 15–20 times per year. He had also had three episodes of ptosis and diplopia in recent one year. These episodes of ophthalmoplegia all occurred at the day the line-shaped headache was remitting and resolved after approximate 7 to 10 days. He had a history of migraine without aura attacks in a frequency of 12–16 times per year for more than 20 years, which remitted 10 years ago. There was a family history of migraine affecting at least 5 individuals across four generations.

**Figure 1:**
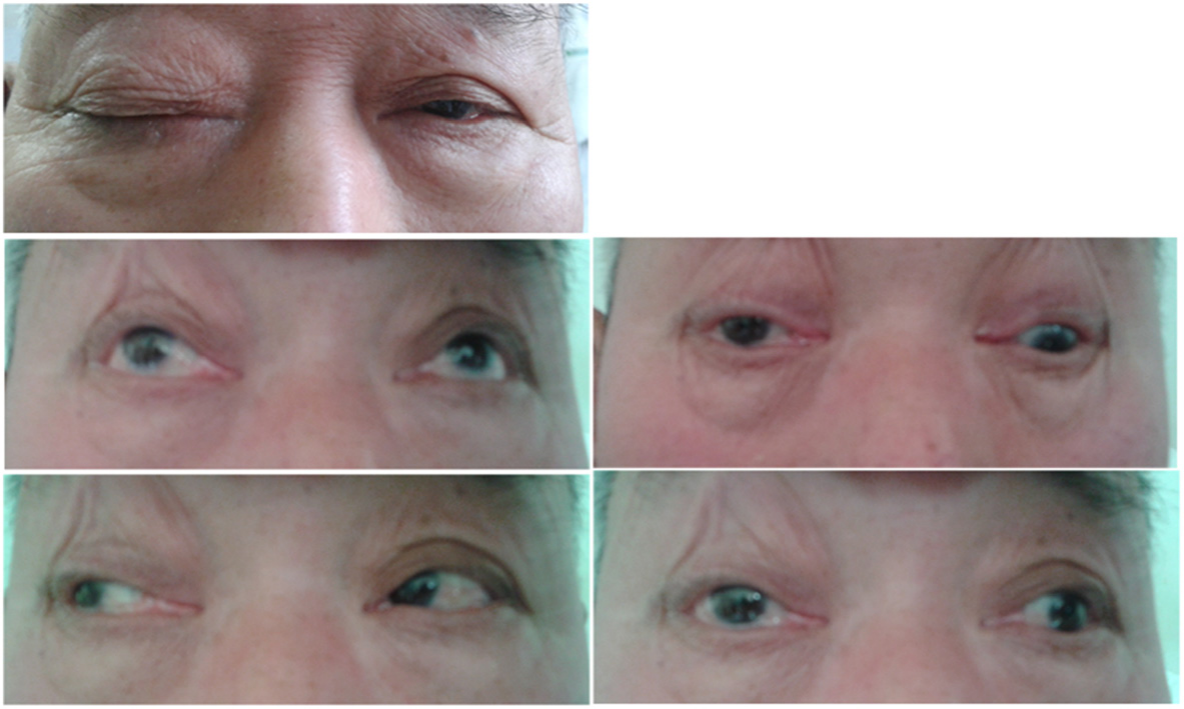
Images of eye showing right ptosis and third cranial nerve palsy.

On examination during the episode of ptosis and diplopia, our patient was found to have a right third nerve palsy with pupil involved, which manifested as an incomplete ptosis and an inability to lower and adduct his right eye. Diplopia was found in all gaze directions, except for looking laterally to the right (Figure 1, taken 3 days after the onset of ophthalmoplegia). His right pupil was little larger than his left, but did react slowly to light and accommodation. Other nervous system examination, including trigeminal nerve examination, was normal.

A brain MRI with contrast was performed 2 days after the onset of ophthalmoplegia, followed by a brain computed tomographic angiography (CTA) conducted 4 days after the onset of ophthalmoplegia, both of which were normal. Cerebrospinal fluid (CSF) analysis performed 3 days after the onset of ophthalmoplegia was normal, including cell counts, protein, glucose levels, and IgG index. Blood routine tests and other investigations including erythrocyte sedimentation rate, vasculitic autoimmune and venereal disease screens were all in normal range.

The patient accepted prophylactic treatment with sodium valproate (500 mg twice a day) together with flunarizine (5 mg twice a day) and he had neither the line-shaped headache nor ophthalmoplegia in the next eight-month follow-up.

## Conclusions

Here we report a patient presenting with a previously undescribed headache which is a paroxysmal head pain restricted in a linear trajectory linking the posterior-parietal region and the ipsilateral eye, and followed by paresis of the third cranial nerve. Apart from the line-shaped pain area similar to that of a novel syndrome epicrania fugax (EF), all other features of the preceding headache are obviously different from those of EF, but apparently similar to those of migraine. The clinical features of this new condition are suggestive of a subtype of OM.

As the pain in our patient is circumscribed in an area of linear shape which is similar to the pain trajectory of EF [7-13], we need to differentiate it from EF. But the much longer duration (days) of motionless distending, pressure-like, and pulsating pain in our paient is dramatically different from the ultrashort duration (less than 10 seconds) of moving stabbing or electric pain in EF patient. And the accompaniments of nausea, phonophobia in our patient’s headache make it obviously different from EF which usually has no accompaniment. On the other hand, the head pain in our patient is obviously different from neuralgia of the supraorbital nerve (SON) and of the greater occipital nerve (GON) though the pain area is correspondent to the scalp area supplied by SON and GON, as the accompaniments of nausea and phonophotophobia in our patient had never been observed in trigeminal (TN) or occipital neuralgia (ON). Lastly, it is unimaginable of a concomitant TN and ON causing an ophthalmoplegia as there is a large longitudinal span in the brain stem from the nucleus of the third cranial nerve to that of SON and GON. Hereby, we can conclude that the head pain in our patient should not be considered as a special EF or a combination of TN and ON associated with ophthalmoplegia.

Based on the ICHD-3 diagnosing criteria for RPON formerly named OM [3]: A. At least two attacks of unilateral headache accompanied by ipsilateral paresis of one, two or all three oculomotor nerves; B. Orbital, parasellar or posterior fossa lesion has been excluded by appropriate investigation; C. Not better accounted for by another ICHD-3 diagnosis, the presentations of our patient could be diagnosed as RPON, though the head pain was distributed in a line-shaped area. According to the current theory that RPON may be initiated by an inflammatory process that affects the oculomotor nerve and irritates the trigeminal sensory fibers, triggering headaches, as sensory fibers of the ophthalmic division of the trigeminal nerve enter the oculomotor nerve [14,15]. Accordingly, it might be speculated that the neuropathy of the third cranial nerve irritates the sensory fibers of the ophthalmic division of the trigeminal nerve causing head pain in the area of SON distribution in our patient. But the head pain in our patient was distributed in the area correspondent to the area innervated by SON as well as by GON, it seems impossible that the parietal and occipital head pain is caused by the irritation of GON due to the third cranial nerve neuropathy, as no sensory fibers of occipital nerve enter the oculomotor nerve and the GON nucleus is far away from the third cranial nerve root and nucleus. Thus, it is implausible for our patient to be diagnosed as RPON as it is hard to explain how the third cranial nerve neuropathy irritates headache circumscribed in a line-shaped area covering the distribution of SON and GON. A diagnosis of OM seems more reasonable as the head pain prior to the ophthalmoplegia had couple of features similar to migraine, such as past history of recurrent migraine attacks, accompaniments of nausea, vomiting, and phonophobia, response to flunarizine and sodium valproate. The term of OM has been replaced by RPON in the ICHD-3 based on the fact that the interval between head pain onset and ophthalmoplegia can extend up to 14 days and focal third cranial nerve enhancement and thickening is frequently demonstrated using MRI; the headache associated with ophthalmoplegia is not always the migraine type and it is not associated with migraine symptoms such as nausea or vomiting; treatment with corticosteroids is beneficial in some patients [14,15]. But, in our patient, the ophthalmoplegia occurred immediately after the migraine-like headache and recovered within 10 days without corticosteroid treatment, and the brain MRI was normal. The clinical features of this new condition are more suggestive of a subtype of OM but not RPON, indicating that a migraine but not a cranial neuropathy mechanism may play a critical role in the ophthalmoplegia starting with a headache circumscribed in a line-shaped area in our patient.

The pathogenesis of the ophthalmoplegia associated with a line-shaped headache in our patient is unknown. It is common for headache patients including migraineurs to localise their pain to a particular area of occipital, parietal, frontal, and orbital region [16]. Recently, there is key experimental data suggesting a probable role of ventroposteromedial (VPM) nucleus of the thalamus in localising a pain to a particular region [17]. However, it is unimaginable that the localization of a line-shaped pain area is integrated just in the thalamus without inputs from a peripheral line-shaped area of nociceptors though the migraine onset does not require the peripheral sensory inputs to activate the trigeminovascular system [18]. The cortical spreading depression (CSD)-induced long-lasting activation of meningeal nociceptors innervated by the fibers of SON or GON [19] has been accepted to be one of the original migraine pathophysiological processes leading to the activation of central trigemino-vascular neurons in the spinal trigeminal nucleus (C1-2) underlying migraine headache [20]. Recently animal studies on familial hemiplegic migraine type 1 (FHM1) model has shown that CSD can readily propagate into the basal ganglia and diencephalon [21,22] causing hemiparesis [23,24]. Hereby, we propose that meningeal nociceptors in a line-shaped area parallel to the superior sagital sinus (SSS) is sensitized due to meningeal immuno-vascular interactions [25] and thus prone to be activated by CSD in our patient, resulting in a line-shaped cephalalgia through migraine pain pathway, and while the CSD propagates into diencephalon it may cause the third cranial nerve palsy. In some conditions, CSD may activate wider area of meningeal nociceptors leading to typical migraine attacks in our patient. This is seemly supported by the history of migraine attacks in our patient.

In summary, we encountered a patient whose clinical presentations met the diagnosing criteria for RPON but were more suggestive of a subtype of OM. This indicates that OM may exist as an entity and some OM may be wrongly grouped under the category of RPON in the international headache classification. More case reports are helpful to better understand the pathophysiological mechanisms of OM.

## Consent

Signed consent is available from the patient for this report publication.

## Abbreviations

RPON: Recurrent painful ophthalmoplegic neuropathy; OM: Ophthalmoplegic migraine; ICHD: International classification of headache disorders; MRI: Magnetic resonance imaging; CTA: Computed tomographic angiography; CSF: Cerebrospinal fluid; EF: Epicranial fugax; SON: Supraorbital nerve; GON: Greater occipital nerve; TN: Trigeminal neuralgia; ON: Occipital neuralgia; CSD: Cortical spreading depression; SSS: Superior sagital sinus; FHM1: Familial hemiplegic migraine type 1.

## Competing interests

The authors declare that they have no competing interests.

## Authors’ contributions

YW interviewed, diagnosed and treated the patients, interpreted the data and drafted the manuscript for content. All other authors listed contributed to the follow-up of the patients and literature reviewing. All authors read and approved the final manuscript.
